# Nitrate Potassa in Chills

**Published:** 1890-06

**Authors:** J. D. Hunter

**Affiliations:** 352 Tulane Avenue; New Orleans, La.


*Editor Daniel's Texas Medical Journal:*

### NITRATE POTASSA IN CHILLS.

NEW ORLEANS, La., May 12, 1890.

*Editor Daniel's Texas Medical Journal:*

DEAR SIR:—To answer fully and specifically the great number of communications received from physicians throughout the country, asking additional information relative to the use of potassa nitras in malarial affections, would necessitate the occupation of the greater part of my time. I will say in brief that I have treated more than two hundred cases of chronic chills of malarial origin, from a few months to years standing; many complicated with enlargement of the liver and spleen, dropsy, jaundice, etc., all more or less, emaciated and anemic. Nearly every case was cured with a single dose of potassa nitras. From two to fifteen grains of the salt, according to age, dissolved in a half ounce of water, administered just prior to the chill or during its continuance, did not only abort the chill, but effectually prevented its recurrence. In order to test the value of the remedy I employed no subsequent treatment, but left the restoration of health (which was in nearly every instance rapid and satisfactory) to the *vis medicatrix naturæ.*

I am fully assured that from two to fifteen grains of potassa nitras will usually abort or arrest a chill arising from any cause. A large dose is not well borne by the stomach, and frequently, in my hands, caused alarming and distressful symptoms, by producing a prolonged depression of the heart's action. I am

Most respectfully,

J. D. HUNTER, M. D.,
352 Tulane Avenue.

